# The Polygonal Cell Shape and Surface Protein Layer of Anaerobic Methane-Oxidizing *Methylomirabilis* *lanthanidiphila* Bacteria

**DOI:** 10.3389/fmicb.2021.766527

**Published:** 2021-12-01

**Authors:** Lavinia Gambelli, Rob Mesman, Wouter Versantvoort, Christoph A. Diebolder, Andreas Engel, Wiel Evers, Mike S. M. Jetten, Martin Pabst, Bertram Daum, Laura van Niftrik

**Affiliations:** ^1^Department of Microbiology, Faculty of Science, Radboud University, Nijmegen, Netherlands; ^2^Living Systems Institute, University of Exeter, Exeter, United Kingdom; ^3^College of Engineering, Mathematics and Physical Sciences, University of Exeter, Exeter, United Kingdom; ^4^Netherlands Centre for Electron Nanoscopy (NeCEN), Leiden University, Leiden, Netherlands; ^5^Department of Bionanoscience, Delft University of Technology, Delft, Netherlands; ^6^Department of Chemical Engineering, Delft University of Technology, Delft, Netherlands; ^7^Department of Biotechnology, Delft University of Technology, Delft, Netherlands; ^8^College of Life and Environmental Sciences, University of Exeter, Exeter, United Kingdom

**Keywords:** *Methylomirabilis*, NC10 phylum, anaerobic methane oxidation, S-layer, cell shape, cryo-tomography, sub-tomogram averaging

## Abstract

*Methylomirabilis* bacteria perform anaerobic methane oxidation coupled to nitrite reduction via an intra-aerobic pathway, producing carbon dioxide and dinitrogen gas. These diderm bacteria possess an unusual polygonal cell shape with sharp ridges that run along the cell body. Previously, a putative surface protein layer (S-layer) was observed as the outermost cell layer of these bacteria. We hypothesized that this S-layer is the determining factor for their polygonal cell shape. Therefore, we enriched the S-layer from *M. lanthanidiphila* cells and through LC-MS/MS identified a 31 kDa candidate S-layer protein, mela_00855, which had no homology to any other known protein. Antibodies were generated against a synthesized peptide derived from the mela_00855 protein sequence and used in immunogold localization to verify its identity and location. Both on thin sections of *M. lanthanidiphila* cells and in negative-stained enriched S-layer patches, the immunogold localization identified mela_00855 as the S-layer protein. Using electron cryo-tomography and sub-tomogram averaging of S-layer patches, we observed that the S-layer has a hexagonal symmetry. Cryo-tomography of whole cells showed that the S-layer and the outer membrane, but not the peptidoglycan layer and the cytoplasmic membrane, exhibited the polygonal shape. Moreover, the S-layer consisted of multiple rigid sheets that partially overlapped, most likely giving rise to the unique polygonal cell shape. These characteristics make the S-layer of *M. lanthanidiphila* a distinctive and intriguing case to study.

## Introduction

*“Candidatus* Methylomirabilis” bacteria are anaerobic methanotrophs that oxidize methane to carbon dioxide coupled to the reduction of nitrite to dinitrogen gas (Ettwig et al., 2010). For a long time, this process was thought to be impossible due to the high activation energy needed to break the C-H bond of methane. However, in 2006 microbe-mediated nitrite-dependent anaerobic oxidation of methane (AOM) was discovered (Raghoebarsing et al., 2006). Nitrite-dependent AOM was attributed to “*Candidatus* Methylomirabilis oxyfera,” a bacterium belonging to the NC10 phylum (Raghoebarsing et al., 2006). Since their discovery, NC10 bacteria have been found in a variety of natural [freshwater (Raghoebarsing et al., 2006; Deutzmann and Schink, 2011; Chen et al., 2015; Yan et al., 2015; Graf et al., 2018), marine (He et al., 2015; Padilla et al., 2016), brackish (Zhang et al., 2018)] and man-made (wastewater treatment plants; Luesken et al., 2011; Ho et al., 2013) ecosystems, indicating that these bacteria might contribute significantly to the global carbon and nitrogen cycles.

*Methylomirabilis* bacteria are cultivated as flocculent enrichment cultures in bioreactor systems. Even though these bacteria grow anaerobically, the postulated pathway for nitrite-dependent methane oxidation still relies on oxygen. Based on genomic analysis and isotope labeling experiments, it was proposed that nitrite is converted to nitric oxide which is then dismutated by a putative NO dismutase (NOD) enzyme (Ettwig et al., 2010, 2012) to nitrogen and oxygen. The oxygen would then be used for methane activation by the particulate methane monooxygenase (pMMO) enzyme. In the end, methane is oxidized to carbon dioxide as end product (Ettwig et al., 2010).

An ultrastructural study of the first enriched and described *Methylomirabilis* species, *M. oxyfera* (Wu et al., 2012), showed that these microorganisms are also unique with respect to their cell morphology. *M. oxyfera* are ca. 300 nm wide and 1.5 μm long diderm bacteria with a polygonal cell shape. The outermost layer of the cell envelope contains multiple longitudinal ridges that end in a cap-like structure at the cell poles. This outermost layer of *M. oxyfera* was proposed to be a surface protein layer (S-layer), about 8 nm thick, of oblique or square symmetry (Wu et al., 2012). Since S-layers, have been described to promote and maintain the cell shape of bacteria, it was postulated that the S-layer of *M. oxyfera* is the responsible factor for its polygonal shape (Wu et al., 2012).

Besides the canonical shapes of cocci, bacilli, filaments and spirals, several unusual cell shapes have been described for bacteria and archaea. Some examples are the flat and square cells of the archaeon *Haloquadratum walsbyi* (Walsby, 1980), the star-shaped bacteria of the genus *Stella* (Vasilyeva, 1985) and the square or triangular archaeal cells of *Haloarcula* species (Nishiyama et al., 1992; Oren et al., 1999). It is often difficult to assess what the evolutionary advantage behind these unusual morphologies is. The most popular hypothesis is that microorganisms need to increase their surface area to volume ratio (SA:V) to balance nutrient uptake through diffusion and disposal of by-products. Any shape more complex than a sphere increases the SA:V compared to a sphere of the same volume (Purcell, 1977; Koch, 1996). However, maintaining a rod or filamentous shape (or any shape other than a sphere) requires the cell to counterbalance the surface tension and the cell osmotic pressure, which would force the cell into a sphere (Young, 2004). For this reason, the evolution of a robust cell envelope and a cytoskeleton are paramount (van Teeseling et al., 2017).

S-layers are an almost universal feature in the cell envelope of Archaea and widespread in Bacteria (Baumeister et al., 1988; Sleytr et al., 1999; Klingl, 2014). In most cases, S-layers consist of a single (glyco)protein that self-assembles into a 2-dimensional, paracrystalline array covering the entire cell surface. S-layer lattices exist in oblique (p1, p2), square (p4), and hexagonal (p3, p6) symmetries, with a center-to-center spacing of the S-layer unit cell between 4 and 35 nm. A high percentage of the S-layer lattice (30–70%) is occupied by pores of varying diameters (Sleytr and Sára, 1997; Sleytr et al., 2014). S-layer proteins have been documented in a wide range of masses, generally between 40 and 200 kDa (Sleytr and Sára, 1997; Sleytr et al., 2014). These proteins are mostly weakly acidic and contain 40–60% hydrophobic amino acids (Sleytr and Sára, 1997). The bonds between the S-layer proteins are usually non-covalent and the attachment to the underlying cell envelope layer is a combination of weak bonds, such as hydrophobic, hydrogen and ionic bonds (Sleytr et al., 2014). However, in the case of diderm Bacteria and Archaea, the underlying cell envelope layer is the lipid bilayer of the outer membrane and of the cytoplasmic membrane, respectively. In monoderm bacteria, however, the S-layer is anchored to the cell wall peptidoglycan or to secondary cell wall polymers (Sleytr et al., 2014; Engelhardt, 2016). S-layer proteins undergo a number of post-translational modifications, among which glycosylation is the most frequent form (Sleytr et al., 2014). Many S-layer proteins are either N- or O- glycosylated, even though in a few cases both types of glycosylation are found on one protein (Schäffer et al., 2001). S-layers have been studied since 1953 (Houwink, 1953) and to date several S-layer proteins have been isolated and characterized. It has become apparent that S-layer proteins show a low degree of conservation on the level of the primary sequence (Sleytr et al., 2014).

S-layers fulfill several functions, most of which are still only hypothetical. Restricting the field to non-pathogenic microorganisms, S-layers can serve as protective coat, molecular sieve, molecule and ion trap; mediate cell adhesion and surface recognition and are involved in the determination of the cell shape (Baumeister et al., 1988; Engelhardt, 2007a,b; Sleytr et al., 2014).

In this study we characterized the cell shape and S-layer (composition and structure) of a recently enriched *Methylomirabilis* bacteria species, *M. lanthanidiphila* (Versantvoort et al., 2018), which similar to *M. oxyfera* exhibits a polygonal cell shape. To study the role of the S-layer in the determining the cell shape of *M. lanthanidiphila*, we used an array of biochemical and cryo-electron microscopy (cryo-EM) methods to enrich, identify, localize, and characterize the S-layer protein and lattice. We find that the S-layer has a hexagonal symmetry and is composed of a single repeating protein unit identified as mela_00855. As other S-layers, the *M. lanthanidiphila* one consisted of individual S-layer sheets. However, unlike other S-layers observed to date, the S-layer sheets of *M. lanthanidiphila* protrude from the outer membrane, detaching from the cell body, and giving rise to the ridges that likely determine the polygonal shape of the cell.

## Materials and Methods

### Enrichment Conditions

*M. lanthanidiphila* (Versantvoort et al., 2018) was cultivated as a flocculent enrichment culture (∼70% *M. lanthanidiphila*) in a 10 L continuous sequencing batch reactor (Applicon Biotechnology BV, Delft, the Netherlands), originally inoculated with sediment samples from a ditch in the Ooijpolder (the Netherlands) (Ettwig et al., 2009). The culture was kept anoxic by a continuous supply of a gas mixture composed of methane and carbon dioxide (95:5, v/v) and the medium was continuously flushed with a mixture of argon and carbon dioxide (95:5, v/v). The temperature was kept stable at 30°C and the system was stirred at 100 RPM. The bioreactor volume was kept at 10 L by a level sensor-controlled pump in sequential cycles of feeding (22.5 h) and resting (30 min to settle, 60 min to pump out excess medium). The HRT (hydraulic retention time) was 10 days and the composition of the medium was 30–40 mM NaNO_2_ (depending on culture performance), 0.78 mM MgSO_4_, 1.96 mM CaCl_2_, and 0.73 mM KH_2_PO_4_ and the following trace elements: 5.4 μM FeSO_4_, 0.26 μM ZnSO_4_, 0.15 μM CoCl_2_⋅6H_2_O, 2.82 μM CuSO_4_, 0.24 μM NiCl_2_, 0.07 μM H_3_BO_3_, 0.3 μM MnCl_2_, 0.05 μM Na_2_WO_4_, 0.12 μM Na_2_MoO_4_, 0.14 μM SeO_2_, and 0.12 μM CeCl_2_, which was adapted from a previously published study (Ettwig et al., 2010).

The presence of *M. lanthanidiphila* was monitored during enrichment by fluorescence *in situ* hybridization (FISH) using a newly developed probe specific for *M. lanthanidiphila* (MLanth181, [TCCCATGAGATCCTCACAGG]), which targets bases 181–200 in the 16 s rRNA gene sequence (NCBI sequence ID CABIKM010000010.1:2618–4176), a region just upstream of the target of the DAMOBACT-0193 probe, which targets all known *Methylomirabilis* species. The MLanth181 probe has 6 mismatches with the *M. oxyfera* sequence (NCBI sequence ID FP565575: 1586103–1587651) as shown using pairwise alignment of the 16 S rRNA gene sequences of *M. oxyfera* and *M. lanthanidiphila*. No additional targets of the MLanth181 probe (Raghoebarsing et al., 2006) could be identified using the SILVA database (Quast et al., 2013).

### Enrichment of the *M. lanthanidiphila* S-Layer Patches

The enrichment of S-layer patches was performed as described previously (van Teeseling et al., 2015). Approximately 60 ml of biomass were harvested from the *M. lanthanidiphila* enrichment culture and gently pottered on ice to disrupt the bacterial aggregates. Cells were concentrated by centrifugation (Allegra X-15R, Beckman Coulter) at 10,000 g for 20 min at 4°C. The supernatant was discarded, and the pellet was resuspended in 4 ml of medium. The sample was boiled in 4% SDS for 1 h. Subsequently, the sample was allowed to cool down and washed three times by ultracentrifugation (Beckman-Coulter Optima-XE-90 ultracentrifuge) at 138,550 g for 10 min at 25°C. Each time the pellet was thoroughly resuspended in 8 ml of MilliQ. To digest the peptidoglycan sacculi, a lysozyme treatment (final concentration 10 mg/ml) was performed overnight at 37°C with vigorous shaking. The lysozyme and peptidoglycan residues were removed from the sample by an additional ultracentrifugation step.

To isolate S-layer suitable for cryo-EM the isolation procedure was further optimized. A sample of 3 ml biomass from the *M. lanthanidiphila* enrichment culture was passed 10 times through a ball-bearing homogenizer (Isobiotech), using a ball with 8 μm clearance, to obtain a homogeneous sample. This sample was boiled for 1.5 h in 4% SDS to liberate the S-layer. S-layers were pelleted and washed 3 times by ultracentrifugation as described above. The resulting pellet was resuspended in 200 μl MilliQ and dialyzed (50 kDA cutoff) against 300 ml MilliQ containing activated biobeads (Biorad) for 2 days at 4°C to adsorb the remaining SDS. Sample quality was tested by transmission electron microscopy using negative stain (2% uranyl acetate) on grids containing a continuous carbon film (200 mesh, copper).

### LC-MS/MS for Identification of the *M. lanthanidiphila* S-Layer Protein and Antibody Generation

Three dilutions of the enriched S-layer patches (undiluted, diluted 1:10 and 1:100 with MilliQ) were processed by in-solution digestion to obtain peptides for LC-MS/MS. Each sample had a total volume of 100 μl. Samples were diluted 1:1 with 8 M urea in 10 mM Tris-HCl pH 8. 1 μl 50 mM DTT was added to the sample and incubated for 20 min at RT. Subsequently, 1 μl of 50 mM iodoacetamide was added and the sample was incubated for an additional 20 min in the dark. LysC enzyme (1 μl, 0.5 μg/μl stock) was added to the sample and incubated for 3 h at RT. Samples were then diluted threefold with 10 mM ammonium bicarbonate and trypsin (1 μl, 0.5 μg/μl stock) was added. Samples were incubated overnight at 37°C and stored at −80°C. Tryptic peptides were desalted and concentrated using C18 solid phase extraction (Omix tips, Agilent Technologies). Peptides were analyzed using nanoflow ultra-high pressure C18 reversed phase liquid chromatography coupled online to a quadrupole orthogonal time-of-flight mass spectrometer (maXis Plus, Bruker Daltonics) via a vacuum assisted axial desolvation nanoflow electrospray ionization source (Captivesprayer, Bruker Daltonics). Peptides were separated using a linear gradient of 5–45% acetonitrile in 0.1% acetic acid for 60 min at a flow rate of 500 nl/min. The mass spectrometer was programmed to acquire 1 MS spectrum at 3 Hz with subsequent data dependent MS/MS spectra at precursor intensity scaled acquisition speeds (3 Hz at 10,000 intensity up to 16 Hz at 150,000 intensity). Total MS + MS/MS duty cycle was 3 s. Dynamic exclusion was enabled to prevent re-analysis of the same precursor ions. Acquired data files were processed in DataAnalysis 4.2 (Bruker Daltonics) to extract MS/MS data for subsequent database searches. Proteins were identified using the MASCOT search tool (Matrix Science, London, United Kingdom) and an in-house protein sequence database of the predicted *M. lanthanidiphila* proteome. MASCOT search parameters included tryptic specificity, maximum of two missed cleavages, Carbamidomethyl (C) as fixed modification, and Oxidation (M) and Deamidation (NQ) as variable modifications. Precursor mass tolerance was set to 10 ppm and fragment ion mass tolerance to 0.05 Da. A single protein was identified by the MS/MS database search which was mela_00855 (NCBI sequence ID: VUZ84482.1). The full protein sequence was handed over to the company GenScript. A protein fragment (33–260 aa) of mela_00855 was synthesized and heterologously expressed in *E. coli*. The protein fragment was then used to raise antibodies in rabbit. The pre-immune serum and the final bleed (crude antiserum) were used for immunoblotting and immunogold localization.

### Cell-Free Extract, PAGE and Immunoblotting

A sample of 500 ml was harvested from the *M. lanthanidiphila* bioreactor and centrifuged at 20,000 g for 10 min at 4°C in a Sorvall centrifuge (Sorvall Lynx 4,000). The pellet was resuspended in 60 ml of 20 mM potassium-phosphate (KPi) buffer at pH 7. The cells were centrifuged again at 20,000 g for 10 min at 4°C. The pellet was resuspended in 60 ml of 20 mM KPi with 2.5 mM EDTA at pH 7 and six protease inhibitor tablets (Pierson Protease Inhibitor, Mini tablets, EDTA-free, Thermo Scientific). Cells were sonicated on ice for 10 min, 5 s on and 5 s off. To remove debris and unbroken cells, the sonicated sample was centrifuged at 10,000 g for 10 min at 4°C. The supernatant of lysed cells was used for further PAGE and immunoblotting.

The cell-free extract was boiled for 7 min in SDS sample buffer (50 mM Tris-HCl buffer pH 6.8 containing 5% β-mercaptoethanol, 2% SDS, 10% glycerol and 0.005% bromophenol blue), and 20 μg protein per lane was loaded onto 4–15% Criterion TGX precast gels (Bio-Rad) for PAGE according to manufacturer’s instructions. After PAGE separation, the proteins were transferred from the gel onto a Trans-blot Turbo, Midi format 0.2 μm nitrocellulose transfer membrane (Bio-Rad) with the Turbo blotter system (Bio-Rad) according to manufacturer’s instructions. The blotting was performed at 2.5 A, 25 V for 7 min. Dried blots were stored at 4°C.

Prior to starting the immunoblot protocol, blots were kept at RT for 30 min and then incubated in MilliQ water for an additional 30 min. Blocking was performed for 1 h in 1% BSA in 10 mM TBS (10 mM Tris-HCl, 137 mM NaCl, 2.7 mM KCl, pH 7.4). The blots were then incubated for 60 min in α-mela_00855 antiserum diluted 125-, 500- or 1,000-fold in blocking buffer. The negative controls were incubated in the pre-immune serum diluted 125-fold or incubated in blocking buffer without primary antibody. The blots were then washed three times for 10 min in TBS containing 0.05% Tween and incubated for 60 min in monoclonal mouse anti-rabbit IgG alkaline phosphatase conjugate (Sigma-Aldrich) diluted 150,000-fold in blocking buffer. The blots were washed two times for 10 min in TBS containing 0.05% Tween and two times for 10 min in TBS. Finally, blots were incubated with a 5-bromo-4-chloro-3-indolylphosphate (BCIP)/nitroblue tetrazolium (NBT) liquid substrate system (Sigma-Aldrich) for 5 min and rinsed for 10 min in MilliQ water.

### Immunogold Localization of the S-Layer Protein in *M. lanthanidiphila* Ultrathin Sections

*M. lanthanidiphila* cells were harvested from the bioreactor and cryo-fixed by high-pressure freezing (Leica HPM 100, Leica Microsystems, Vienna, Austria). Samples were placed into a 100-μm cavity of a type-A platelet (3 mm diameter; 0.1–0.2-mm depth, Leica Microsystems) and closed with the flat side of a lecithin-coated type B platelet (3-mm diameter; 0.3-mm depth). The frozen samples were stored in liquid nitrogen.

For Lowicryl HM20 embedding, frozen samples were freeze-substituted in 0.2% uranyl acetate in acetone. The substitution started at −90°C for 48 h; brought to −70°C at 2°C per hour and kept at −70°C for 12 h; brought to −50°C at 2°C per h and kept at −50°C for 12 h in a freeze-substitution unit (AFS2; Leica Microsystems, Vienna, Austria). To remove unbound uranyl acetate, the samples were washed twice with 100% acetone for 30 min at −50°C. Keeping the temperature stable at −50°C, the sample was infiltrated with a graduated series of Lowicryl HM20 (10, 25, 50, and 75%) in acetone. Each step was 1 h long. Three final infiltration phases were performed with 100% Lowicryl HM20: first for 1.5 h, then overnight and finally 2 h. Polymerization of the resin was obtained by irradiating the sample with UV light for 120 h at −50°C after which the temperature was brought to 0°C in 24 h at 2.1°C per hour. UV light was switched off and the temperature was increased to 20°C in 5 h at 4°C per hour. Ultrathin sections of 55 nm were cut using a Leica UCT ultramicrotome (Leica Microsystems, Vienna, Austria) and collected on carbon-Formvar-coated 100 mesh copper grids (Agar Scientific).

Grids containing ultrathin sections were rinsed for 10 min in 0.1 M PHEM (1X PHEM contains: 60 mM PIPES, 25 mM HEPES, 10 mM EGTA, and 4 mM MgSO_4_^⋅^7H20) buffer pH 7 and blocked for 20 min in 1% BSAc (Aurion) in PHEM buffer. Grids were incubated for 60 min at RT with the primary antiserum targeting the S-layer protein diluted 1:400 in blocking buffer. Negative controls were incubated for 60 min in blocking buffer without primary antiserum and with the pre-immune serum diluted 1:400. After this incubation, the grids were washed for 10 min in 0.1% BSAc in 0.1 M PHEM buffer and incubated for 30 min with protein A coupled to 10 nm gold particles (PAG 10, CMC UMC Utrecht), diluted 1:60 in blocking buffer. The grids were then washed again first in 0.1% BSAc in 0.1 M PHEM buffer for 5 min and then in 0.1 M PHEM buffer for 5 min. To fix the labeling, the grids were incubated for 5 min in 1% glutaraldehyde in 0.1 M PHEM buffer and subsequently washed for 10 min in MilliQ. Post-staining was performed by incubating for 20 min on drops of 2% uranyl acetate, after which the grids were quickly washed in 4 drops of MilliQ and allowed to air dry. The grids containing labeled ultrathin sections were investigated at 60 kV in a JEOL (Tokyo, Japan) JEM-1010 TEM.

### Immunogold Localization of the *M. lanthanidiphila* S-Layer Patches

A sample of 3.5 μl of enriched S-layer patches (obtained as described in the section “Enrichment of the *M. lanthanidiphila* S-layer patches”) was spotted on carbon-Formvar-coated 200 mesh copper grids (Agar Scientific) and incubated for 15 min at RT under moisture-controlled conditions. Subsequently the grids were blotted to remove excess sample and were allowed to air dry overnight. Prior to starting the immunogold localization protocol, the grids were heated at 120°C for 20 min. The grids were rinsed for 6 min on drops of 0.1 M PHEM buffer pH 7 and blocked with 1% BSAc (Aurion) in 0.1 M PHEM buffer pH 7 for 5 min. The incubation with primary antiserum targeting the S-layer protein was performed with a 1:40 dilution in blocking buffer for 25 min. Negative controls were incubated for 25 min in blocking buffer without primary antiserum and with the pre-immune serum diluted 1:40. Grids were rinsed for 6 min in 0.1 M PHEM containing 0.1% BSAc and then incubated for 30 min with the protein A coupled to 10 nm gold particles (PAG 10, CMC UMC Utrecht), diluted 1:60 in blocking buffer. To remove BSAc, the grids were washed in 0.1 M PHEM and fixed with 1% glutaraldehyde in 0.1 M PHEM buffer. The glutaraldehyde was removed with 10 washes with MilliQ. The labeled S-layer patches were stained with 2% uranyl acetate in 0.1% acetic acid for 1 min. The excess staining solution was blotted off and grids were allowed to air dry. The grids containing labeled negative-stained S-layer patches were investigated at 60 kV in a JEOL (Tokyo, Japan) JEM-1010 TEM.

### Cryo-Tomography

#### Whole Cell Cryo-ET

**FIGURE 1:**
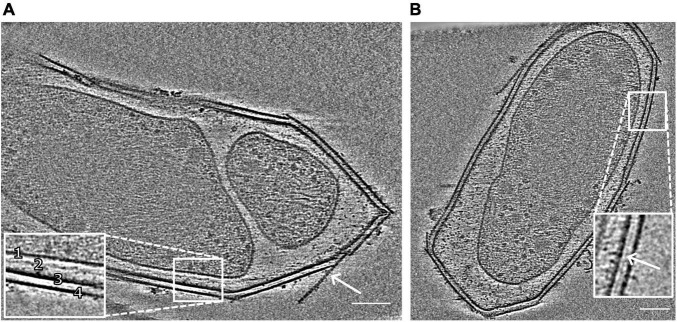
Snapshots of electron cryo-tomograms of two *M. lanthanidiphila* cells. The inset in **(A)** shows the cell envelope (1, cytoplasmic membrane; 2, peptidoglycan layer; 3, outer membrane; 4, S-layer). The peptidoglycan layer is not always visible. The arrow shows an S-layer sheet protruding from the cell. The inset in **(B)** shows a layer (white arrowhead) between the outer membrane and the S-layer. Scale bars 100 nm.

**FIGURE 2:**
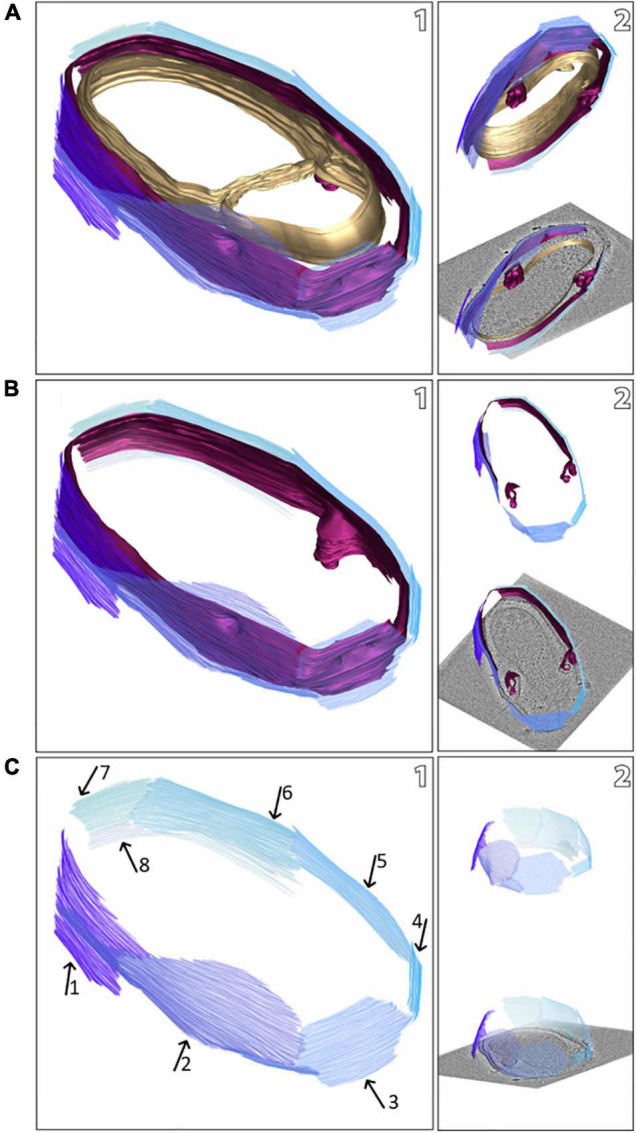
Snapshots of a model of a *M. lanthanidiphila* cell (based on the electron cryo-tomogram in Figure 1B). The S-layer patches are depicted in shades of blue, the outer membrane in magenta, and the cytoplasmic membrane in gold. The complete cell envelope (S-layer, outer membrane, and cytoplasmic membrane) is shown in **(A1)**, the S-layer and the outer membrane are shown in **(B1)**, and the S-layer only (with arrows pointing at different patches) is shown in **(C1)**. **(A2,B2,C2)** are different views of **(A1,B1,C1)**, respectively.

For the whole cell cryo-ET (Figures 1, 2 and Supplementary Movies 1, 2), a sample of 15 ml was collected from the *M. lanthanidiphila* enrichment culture and centrifuged (Beckman Coulter) for 2 min at 5,000 g. The pellet was resuspended in 1 ml of bioreactor medium. Cells were bead-beaten using glass beads (0.5 mm, BioSpec) for 1 min at 50/s oscillation in a tissuelyser (Tissuelyser LT, Quiagen) to break the cell aggregates. To collect the single cells, the sample was pulse-centrifuged in a tabletop microcentrifuge. The supernatant was diluted 1:2 with reactor medium until an OD600 of 0.45.

**FIGURE 3:**
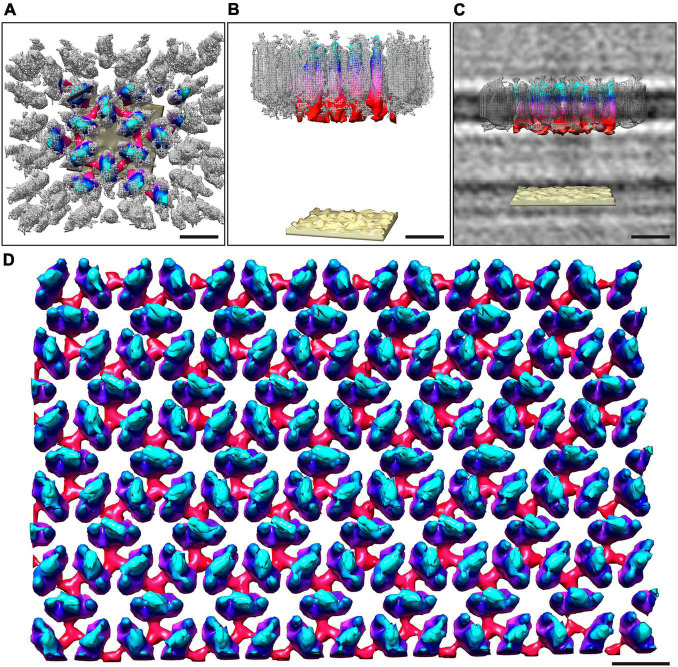
Overlay of the sub-tomogram average obtained from isolated S-layer patches and whole-cell electron cryo-tomography. The map obtained from the whole cell cryo-tomogram is colored by proximity to the cell outer membrane (cyan, membrane-distal; red, membrane-proximal; sand, outer membrane), whereas the sub-tomogram average obtained from isolated S-layer patches is in gray mesh. Both the outward-facing **(A)** and the side views **(B)** show a good correlation between the two models. **(C)** Shows the side view with the median filtered S-layer model after scaling the height of both models to fit the height of the S-layer as observed in the cryo-tomograms. Visual rendering **(D)** of an S-layer sheet after median filtering (6 iterations). The height of the S-layer has been scaled to 7 nm as observed from perpendicular cross-sections in the cryo-tomograms. Scale bars 10 nm.

Prior to sub-tomogram averaging of S-layers *in situ* (Figure 3), the samples were reproduced, under slightly different conditions as before. 3 ml biomass from the *M. lanthanidiphila* bioreactor was passed 3 times through a ball-bearing homogenizer (Isobiotech) using a ball with 8 μm clearance, to break the cell aggregates.

For both datasets, ∼2 μl containing *M. lanthanidiphila* single cells and 0.5 μl 10 nm protein A gold (CMC, UMC Utrecht, the Netherlands) were applied on a glow-discharged Quantifoil type R2/2 copper 200-mesh grids. The cells were frozen in liquid ethane using a Vitrobot Mk4 (FEI, Thermo Fisher Scientific) with controlled humidity (100%) using ablotforce of 1 and a blot time of 3 s. Tilt series were recorded at the Netherlands Center for Electron Nanoscopy (NeCEN, Leiden, the Netherlands) on a Titan Krios (FEI, Thermo Fisher Scientific) microscope at 300 kV equipped with a Schottky type field emission gun and a K3 camera (Gatan) operated in counting mode.

Tilt series were acquired using UCSF Tomo (Zheng et al., 2007). Initially, a linear tilt scheme from −60^°^ to + 60^°^ with a 2^°^ increment at −6 μm defocus and a magnification of 26,000× (pixel size of 4.6 Å) was employed. To improve data quality for sub-tomogram averaging, data were collected at 33,000× magnification (pixel size of 2.64 Å) and dose-symmetric tilt scheme (Hagen et al., 2017) from −60 to + 60 with a 2° increment at −4 μm defocus was employed using SerialEM (Mastronarde, 2005). The total dose on the record area did not exceed 100 e-/Å2 and varied between 93 and 96.5 e^–^/Å^2^. Tomograms were reconstructed and CTF corrected in IMOD (Kremer et al., 1996) and binned threefold (final pixel size of 9.267 Å for whole cell cryo-ET; pixel size of 7.92 Å, for sub-tomogram averaging). Tomograms were either reconstructed using SIRT (simultaneous iterative reconstruction technique) to optimize contrast or weighted back projection for sub-tomogram averaging. The whole cell movies (Supplementary Movies 1, 2) were made in IMOD.

#### Cryo-ET of Isolated S-Layers and Sub-Tomogram Averaging

**FIGURE 4:**
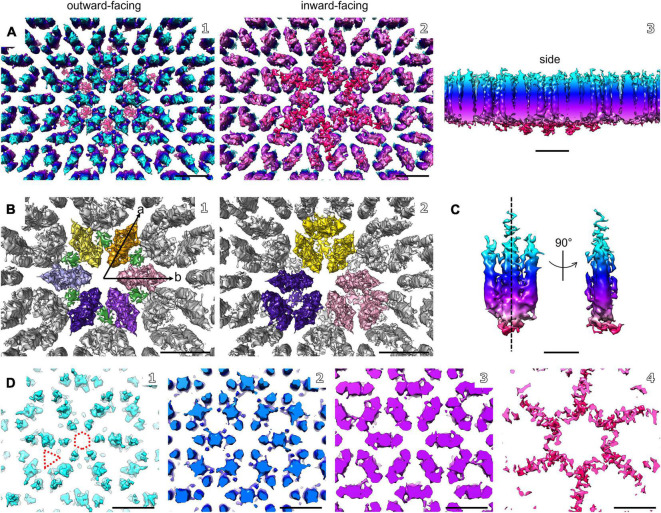
Electron cryo-tomography and sub-tomogram averaging of *M. lanthanidiphila* S-layer. **(A)** Sub-tomogram average of isolated *M. lanthanidiphil*a S-layer patches. Maps are colored by proximity to the cell outer membrane (cyan, membrane-distal; red, membrane-proximal), as in **(C,D)**. **(B)** in **(B1)** putative dimers of mela_00855 as the S-layer building block are highlighted in different colors, and the densities projecting toward the outer membrane are in green. The unit cell dimensions are 11.9 × 11.6 nm (a and b arrows, respectively) including an angle of 60°. **(B2)** shows the staggered conformation of the repeating unit-cell within the S-layer. **(C)** shows the isolated putative dimer of mela_00855. The structure is symmetrical along the dashed line. **(D)** slices through the sub-tomogram average from membrane-distal **(D1)** to membrane-proximal **(D4)**. The dashed hexagon and triangle in **(D1)** highlight the hexagonal and triangular pores. Scale bar in **(A,B, and D)** is 10 nm; scale bar in **(C)** is 5 nm.

For cryo-ET of isolated S-layers and sub-tomogram averaging (Figure 4), isolated S-layers were obtained as described above (section “Enrichment of the *M. lanthanidiphila* S-layer patches”). Before plunge freezing, potential aggregates in the sample were pelleted and 2 μl of the supernatant was mixed with 0.5 μl 10 nm ProteinA gold solution (CMC, UMC Utrecht) on glow discharged Quantifoil type R2/2 copper 200-mesh grids. Grids were plunge frozen in liquid ethane using a Vitrobot Mk4 (FEI/Thermo Fisher Scientific) with controlled humidity (100%) using a blot force of 2, and a blot time of 2.5–3 s. Tilt series were recorded at the Netherlands Centre for Electron Nanoscopy (NeCEN, Leiden, Netherlands) using a Titan Krios (FEI, Thermo Fisher Scientific) microscope at 300 kV equipped with a Schottky type field emission gun and a K3 camera (Gatan) operated in counting mode. The tilt series were acquired in low-dose using SerialEM (Mastronarde, 2005), employing a dose-symmetric tilt-scheme (Hagen et al., 2017) from −60^°^ to + 60^°^ with a 2^°^ increment at −4 μm defocus. The total dose on the record area did not exceed 100 e-/Å2. Recorded tilt series were reconstructed using IMOD (Kremer et al., 1996), and tomograms were generated using the weighted back-projection algorithm.

### Sub-Tomogram Averaging

To obtain the structure of the S-layer *in situ* and in its isolated form, sub-volumes were initially selected by applying a random grid of points over the S-layer patches in 3Dmod (IMOD software package). Using PEET (Nicastro et al., 2006; Heumann et al., 2011), sub-volumes were extracted, aligned, and averaged. A total of 8,938 sub-volumes of 120 × 120 × 60 pixels in dimension were used for averaging the isolated S-layer patches and 1,330 sub-volumes for obtaining the average from the S-layer *in situ*. P6 symmetry was applied to the final average obtained from unbinned and unfiltered tomograms. The final S-layer average was visualized and segmented using UCSF Chimera (Pettersen et al., 2004). A resolution of 21 Å nm of the average was estimated based on the reflections in its power spectrum calculated by IMOD (Kremer et al., 1996). Supplementary Movie 3 was made using UCSF Chimera.

### S-Layer Sequence Coverage and Protein Glycosylation Analysis

#### In-Solution Digestion of Isolated S-Layer Protein Enrichment

A volume of 50 μl of S-layer enrichment sample (approx. 0.1 mg/ml protein content) was diluted 1:1 with 8 M Urea in 10 mM Tris-HCl buffer pH 8.0. To this solution, 1 μl of freshly prepared 50 mM DTT solution was added and incubated at 37°C for 1 h. Next, 1 μl of a freshly prepared 50 mM IAA (iodoacetamide) solution was added and incubated in the dark for 30 min. The solution was diluted to below 1 M Urea using 200 mM bicarbonate buffer and one aliquot protein each were digested using sequencing grade Trypsin or Chymotrypsin, respectively (Promega) at 37°C overnight (protease/protein of approx. 1:50). Finally, the protein digests were desalted using an Oasis HLB 96 well plate (Waters) according to the manufacturer protocols. The purified peptide fraction was speed-vac dried and stored at −20°C until further processed.

#### Shotgun Proteomic Analysis

The speed-vac dried peptide fraction was resuspended in 3% acetonitrile and 0.1% formic acid. An aliquot corresponding to approx. 250 ng protein digest was analyzed using a one-dimensional shotgun proteomics approach. Thereby, 1 μl of the protein digest was analyzed using a linear gradient from 5 to 30% B over 40 min, and further to 60% B over 15 min, maintaining a flow rate of 350 nL/min. MS1 level scans were performed over the mass range from 385 to 1250 m/z at 70 K resolution with an AGC target of 3 e6 and a max IT of 100 ms. Top10 DDA fragmentation spectra were acquired at 17.5 K resolution, with an AGC target of 2e5, a max IT of 54 ms and by using a NCE of 28. Unassigned, singly charged as well as 6, 8, and > 8 charged mass peaks were excluded.

#### Database Search and Data Processing

Raw data were analyzed using PEAKS Studio X (Bioinformatics Solutions Inc., Canada) allowing 20 ppm parent ion and 0.02 Da fragment mass error tolerance. Search conditions further included considering 4 missed cleavages, carbamidomethylation as fixed and methionine oxidation and N/Q deamidation as variable modifications. Semi-specific cleavages were allowed. Data were matched against a *M. lanthanidiphila* in-house annotated protein database (Genbank, VUZ84482.1). Database search included the GPM crap contaminant database^1^ and a decoy fusion for determining false discovery rates. Peptide spectrum matches were filtered against 1% false discovery rate (FDR), or a −10lgP peptide score of −41.3, respectively.

### Sequence Analyses

The sequence alignment was performed with the Praline server (Heringa, 1999).^2^

The sequence analysis for glycosylation sites was performed with the GlycoPP v1.0 server (Chauhan et al., 2012).^3^

### Data Deposition

The sub-tomogram averaging electron density maps of the whole cells and isolated S-layer have been deposited in EMDB with accession code EMD-13672 and EMD-13670, respectively. Raw cryo-tomography data have been deposited in EMPIAR with accession code EMPIAR-10822 for the isolated S-layer patches and EMPIAR-10829 for the whole cells. The mass spectrometry proteomics data have been deposited to the ProteomeXchange Consortium^4^ via the PRIDE partner repository with the dataset identifier PXD029319.

## Results

### Cell Structure of *M. lanthanidiphila* Bacteria

**FIGURE 5:**
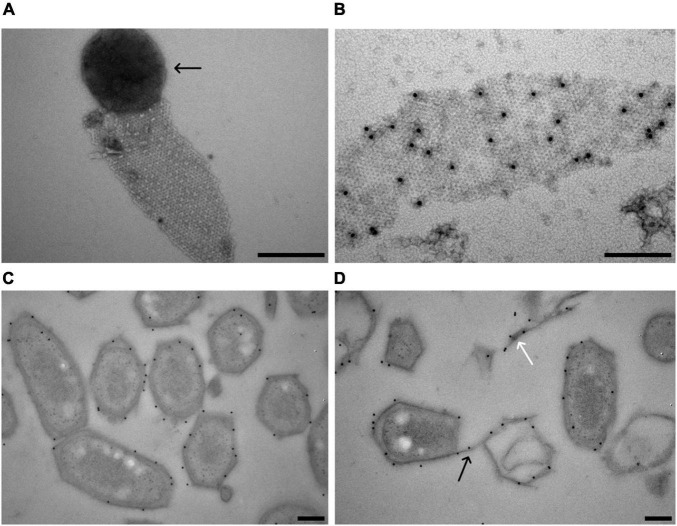
Localization of the *M. lanthanidiphila* S-layer protein. **(A)** Transmission electron micrograph of a negative-stained *M. lanthanidiphila* S-layer patch. A round peptidoglycan sacculus (arrow) is still attached to the S-layer patch. **(B)** Transmission electron micrograph of negative-stained *M. lanthanidiphila* S-layer patch with immunogold labeling (black dots) of the S-layer protein mela_00855. **(C,D)** Transmission electron micrographs of high-pressure frozen, freeze-substituted and Lowicryl-embedded *M. lanthanidiphila* cells showing immunogold localization of the S-layer protein mela_00855. In **(D)** the labeling is present also on the S-layer edges protruding from the cell (black arrow) and on shed S-layer patches (white arrow). Scale bars 200 nm.

The cell shape and cell plan of *M. lanthanidiphila* were investigated by cryo-ET on whole cells. Our data revealed the overall polygonal shape of the *M. lanthanidiphila* cell (Figure 1; Supplementary Movies 1, 2). The S-layer was clearly discernable as a distinct layer ∼ 11 nm above the outer membrane. Instead of adhering to the cell body as usually seen in other microorganisms (van Teeseling et al., 2014; Bharat et al., 2017; Gambelli et al., 2019; Gaisin et al., 2020; von Kügelgen et al., 2020; Oatley et al., 2020. For examples of reviews see Albers and Meyer (2011); Pavkov-Keller et al. (2011), Rodrigues-Oliveira et al. (2017), and Pum et al. (2021), the S-layer of *M. lanthanidiphila* formed several distinct patches. These patches had the appearance of planar sheets and intersected at sharp ridges, thereby likely defining the polygonal shape of the cell. The distance between the S-layer and the outer membrane remained constant, indicating that the S-layer proteins are anchored to the outer membrane, but not to the cytoplasmic membrane that did not have a polygonal shape. The peptidoglycan layer also did not have a polygonal shape. In intact cells (Figure 1A and Supplementary Figure 1) the peptidoglycan appeared to slightly protrude toward the edges of the polygon, without however strictly assuming a polygonal shape. Furthermore, in isolated peptidoglycan sacculi (Figure 5A) the peptidoglycan was round. An additional electron dense layer was present between the outer membrane and the S-layer in all observed *M. lanthanidiphila* cells (Figure 1B, inset). At the ridges, the S-layer sheets protrude, overlapping with each other, and partially detaching from the outer membrane (Figure 1A, arrow). The inset in Figure 1A shows a snapshot of the cell envelope elements: cytoplasmic membrane, peptidoglycan layer, outer membrane, and S-layer. The segmentation of the *M. lanthanidiphila* cell in Figure 1B showed that the S-layer was arranged around the cell in multiple sheets of different sizes that partially overlapped with each other (Figure 2).

### Identification of the *M. lanthanidiphila* S-Layer Protein

Several established protocols were extensively tested and optimized to enrich or isolate the *M. lanthanidiphila* S-layer protein. Specifically, we changed the pH of the solution (low pH, acid treatment), added detergent (Triton X-100), denaturing agents (urea, guanidine hydrochloride, lithium chloride), or chelating agents (EDTA). In almost all cases, the S-layer was released from the cell, but could not be disassembled and did not show any protein bands on SDS-PAGE. Therefore, *M. lanthanidiphila* cells were boiled in 4% SDS and subsequently ultracentrifuged to enrich S-layer patches. The patches were digested in-solution and analyzed for protein content by LC-MS/MS. Only one protein was retrieved that matched to the predicted proteome of *M. lanthanidiphila*. This protein was mela_00855 (gene length 950 bp, protein length 316 aa, MW 31.6 kDa, pI 8.42, Supplementary Table 1). This protein was highly transcribed; 1,539.33 RPKM value, which was in the range of other important proteins such as 1,328.26 for PmoB beta subunit and 6,254.36 for the putative NO dismutase (Nod2, mela_02434) (unpublished data). The protein mela_00855 was annotated as hypothetical protein based on Blastp analysis. The only protein that matched mela_0855 in Blastp analysis was an *M. oxyfera* protein annotated as “exported protein of unknown function” (NCBI sequence ID: CBE67388). The two proteins shared 40.88% protein sequence identity and 97% coverage (Supplementary Figure 2). The amino acid sequence of mela_00855 (Supplementary Table 1) was analyzed for the presence of conserved regions consulting InterProScan (Jones et al., 2014). The protein sequence had two transmembrane regions, one being a predicted N-terminal signal peptide (1–34 aa), and the other a predicted C-terminal IPTL-CTERM protein sorting domain (285–312 aa).

To confirm the identity of the *M. lanthanidiphila* S-layer protein, antibodies were raised against a synthesized protein fragment (from 33 to 260 aa of mela_00855). The affinity and specificity of the crude antiserum was tested and confirmed by immunoblot analysis using *M. lanthanidiphila* cell extracts (Supplementary Figure 3). The S-layer sheets were isolated from *M. lanthanidiphila* cells (Figure 5A) and the mela_00855 antiserum was used to localize the S-layer on negative-stained enriched S-layer patches and thin-sections of resin-embedded *M. lanthanidiphila* cells. The immunogold localization on negative-stained enriched S-layer patches showed abundant and specific binding of the antiserum to the patches (Figure 5B). The immunogold localization on thin sections resulted in the specific labeling of the *M. lanthanidiphila* cell envelope and it was particularly abundant in the area occupied by the outer membrane and the S-layer (Figures 5C,D). Labeling was also present on edges protruding from the bacterial cell wall and on shed S-layer sheets (Figure 5D).

### Structure of the *M. lanthanidiphila* S-Layer

The structure of the *M. lanthanidiphila* S-layer was investigated by cryo-ET and sub-tomogram averaging of enriched S-layer patches (Figure 4, Supplementary Figure 4, and Supplementary Movie 3). The final map was achieved by averaging 8,938 sub-volumes (Figure 4A) and had a resolution of 21 Å based on the Fast Fourier Transform (FFT) power spectrum (see Supplementary Figure 5 for both FFT and FSC). Further, analysis of the FFT power spectrum of the averaged S-layer volumes showed that the S-layer had a hexagonal, p6 symmetry, and the unit cell dimensions were ∼11.6 ×∼11.9 nm including an angle of 60° (Figure 4B1). A single S-layer building block is shown in Figure 4C. This density showed a fork-like appearance, with three extracellular protrusions that join into a single stalk at the inward-facing side of the lattice. When observed in cross sections (Figure 4D) the S-layer building block appeared to have two-fold symmetry with respect to its long axis, which may suggest that the subunit is a dimer. Three S-layer putative dimers constitute the repeating unit cell of the S-layer (Figure 4B2). The S-layer lattice contained two main pore types: a ∼12 nm^2^ hexagonal pore surrounded by six units, and a ∼3.9 nm^2^ triangular pore surrounded by three units (Figure 4D1). On the inward-facing side of the S-layer, the three protein densities surrounding a triangular pore converged and connected at the center of it. As each of the triangular pores has six triangular neighbors and each dimer-like unit is shared between two neighboring triangular pores, the entire S-layer is interconnected. The S-layer patches were roughly perpendicular to the electron beam during data acquisition and, therefore, the map was stretched in Z due to the missing wedge. From the cryo-tomograms of whole *M. lanthanidiphila* cells it could be determined that the S-layer has a thickness of ∼7 nm. For visualization purposes a median filter (6 iterations) was applied to the obtained average and the Z-scaling adjusted to match the thickness obtained from the cryo-tomograms. Multiple copies of the average were aligned to simulate the structure of the S-layer sheet (Figure 3D).

To determine the orientation of the obtained S-layer model with respect to the cell envelope, cryo-ET and subsequent sub-tomogram averaging was performed on whole *M. lanthanidiphila* cells. The *in situ* average of the S-layer confirmed that the fork-like protrusions of the S-layer units face the extracellular environment, while the densities that connect the units with each other face the outer membrane (Figures 3A–C).

S-layer proteins are usually highly glycosylated. To investigate, if this is also the case for mela_00855, we analyzed its primary sequence for N- or O-linked glycosylation sequons using GlycoPP v1.0 (Chauhan et al., 2012). The server predicted 46 putative O-linked and 4 putative N-linked glycosylated sites (Supplementary Table 1). To confirm this prediction, we performed an in-depth characterization of the purified S-layer by high-resolution shotgun proteomics (Supplementary Figure 6). Apart from the C-terminal tail, the complete amino acid sequence could be covered with peptide fragments. However, interestingly no indications for N or O-linked glycans could be found.

## Discussion

We analyzed the cell shape and S-layer structure of *M. lanthanidiphila* to understand the origin of its polygonal cell shape. To this end, we enriched, identified, localized, and characterized the S-layer protein and lattice. The building block of the S-layer was identified as the protein mela_00855. This protein assembled in planar sheets of hexagonal (p6) symmetry. The S-layer sheets partially overlapped and entirely covered the outer membrane. The polygonal shape was clearly observed in both the S-layer and the outer membrane, but not in the peptidoglycan and cytoplasmic membrane, leading us to conclude that the S-layer is very likely responsible for the polygonal shape of this bacterium. This S-layer is highly stable and resilient. In fact, we were unable to break the S-layer patches down to its monomers. Extensive attempts using different protocols were made to disrupt the S-layer protein-protein interactions, but always resulted in the enrichment of S-layer patches and not of single subunits visible on SDS-PAGE. Also in other instances, S-layers have been shown to be impervious to disassembly, for example archaea of the *Thermoproteus* (Messner et al., 1986), *Pyrobaculum* (Phipps et al., 1990, 1991) and *Staphylothermus* (Peters et al., 1995, 1996) genera. However, this resilience is unusual for bacterial S-layers and indicates that this lattice is highly stable, suggesting that the protein-protein interaction occurring between these S-layer proteins can be very strong and possibly of kinds not usually found in interprotein interactions in S-layer lattices, such as covalent bonds.

The S-layer is constituted by several mostly straight patches, distributed with different orientations in order to cover the cell entirely. Moreover, the sheets protrude from the outer membrane toward the extracellular environment still maintaining their orientation. From these observations we concluded that these S-layer sheets are very rigid. A dense network of protein bridges links the S-layer morphological units. These proteinaceous connections are present over every triangular pore throughout the S-layer lattice. We hypothesize that the dense net of protein bridges, together with perhaps stronger protein-protein interactions, establishes the observed rigidity of the S-layer. S-layers are typically highly interconnected structures, and protein connections between the morphological units have been observed in other lattices (Phipps et al., 1990; Pum et al., 1991; Lupas et al., 1994; Arbing et al., 2012; Bharat et al., 2017; Gambelli et al., 2019). Future experiments to probe the rigidity and elasticity of this S-layer could include Atomic Force Microscopy (AFM) to analyze the binding forces between S-layer proteins both on the cell and on enriched S-layer patches (Toca-Herrera et al., 2004; Martín-Molina et al., 2006; Scheuring and Dufrene, 2010; Viljoen et al., 2020).

As opposed to the cytoplasmic membrane and peptidoglycan, the outer membrane followed the polygonal cell shape and cryo-ET revealed an additional layer between the S-layer and outer membrane. A similarly weak electron density between the S-layer and the outer membrane has been observed in *C. crescentus*, another Gram-negative bacterium (Bharat et al., 2017). In this bacterium, the S-layer protein RsaA is attached to the outer membrane via the N-terminus region of the S-layer protein that interacts with the LPS (Ford et al., 2007; von Kügelgen et al., 2020). We thus hypothesize that a similar mechanism could mediate the attachment of the *M. lanthanidiphila* S-layer protein to the outer membrane, via the N-terminus region of mela_00855.

Based on the observations that the outer membrane and the S-layer are the only polygonal components in the cell wall of *M. lanthanidiphila*, we concluded that the S-layer is likely to be responsible for the unusual cell shape of these bacteria. However, additional elements could contribute to the cell shape determination. Particularly, protein filament systems have been shown to be widespread in prokaryotic cells and play a major role in cell shape determination (Wagstaff and Löwe, 2018). Protein filaments such as crescentin and bactofilins are localized in the cytoplasm and are membrane-bound (Ausmees et al., 2003; Deng et al., 2020). Because the inner membrane of *M. lanthanidiphila* does not follow the sharp angles of the outer membrane and S-layer, we exclude that cytoplasmic elements have a structural role in the cell shape maintenance of these bacteria. However, other cytoskeletal elements of still unknown nature could function as a scaffold for the S-layer.S-layer proteins in different microorganisms share little to no similarity (Engelhardt and Peters, 1998; Bharat et al., 2021), sometimes even in the case of closely related microorganisms (Avall-Jääskeläinen and Palva, 2005; Fagan and Fairweather, 2014). The *M. lanthanidiphila* mela_00855 had no homologs (after BLASTp) besides a 40.88% protein sequence identity with a protein from *M. oxyfera* (NCBI sequence ID: CBE67388). The power spectrum of the surface of freeze-etched *M. oxyfera* cells showed its S-layer to have either an oblique (p2) or square (p4) symmetry, and a center-to-center spacing of the S-layer morphological unit of ∼7 nm (Wu et al., 2012). These symmetries are different from what we observed for *M. lanthanidiphila*. Nevertheless, both *M. lanthanidiphila* and *M. oyfera*, have a similar polygonal cell shape and the S-layer occurs as straight patches that partially overlap and protrude from the edges of the cell. It would be very interesting to investigate and compare the S-layers of these two *Methylomirabilis* species in the future.

The mela_00855 protein sequence showed 46 putative O-linked and 4 putative N-linked glycosylated sites. Moreover, the genome organization around mela_00855 shows genes involved in glycosylation (glycosyl transferase, mela_00856, mela_00851; UDP-N-acetyl-D-glucosamine dehydrogenase, mela_00857). Therefore, an in-depth characterization of the enriched S-layer by high-resolution shotgun proteomics was performed aiming for high amino acid sequence coverage. Except for the C-terminal tail, the complete amino acid sequence could be covered with peptide fragments. Thereby, no indications for N- or O-linked glycans were found. Nevertheless, because peptides from the C-terminal tail could not be detected, we cannot exclude the presence of glycosylation in this area (even though preliminary gels and staining attempts seem to exclude this scenario, data not shown). Nevertheless, the C-terminal part includes a very hydrophilic sequence region, and therefore is difficult to capture by the applied analysis approach. Glycosylation is a frequent post-translational modification in S-layer proteins (Ristl et al., 2010; Sleytr et al., 2014), albeit there have been reports of S-layer proteins with limited sugar content (Peters et al., 1987) as well as its absence (Masuda and Kawata, 1983; Li et al., 2018). Glycans have been hypothesized to favor protein stability (Mengele and Sumper, 1992; Engelhardt and Peters, 1998; Li et al., 2020). Interestingly, however, despite the apparent lack of glycosylation, the S-layer of *M. lanthanidiphila* shows remarkable resilience to disassembly. In the case of *H. volcanii* it was shown that environmental conditions (particularly salinity) modulate the glycan structure of the S-layer protein as well as the glycosylation sites (Guan et al., 2011). Therefore, it could be speculated that under different growth conditions glycosylation might be present on the *M. lanthanidiphila* S-layer.

We identified mela_00855 as the only component of the *M. lanthanidiphila* S-layer. Nonetheless, we cannot exclude the possibility that other proteins may be associated to the lattice. Infact, due to the impervious nature of disassembly, the S-layer patches were boiled in SDS prior to LC-MS/MS, likely causing the loss of less resilient protein components (such as membrane-associated proteins). Additionally, no other S-layers have been characterized from members of the NC10 phylum, thereby limiting our understanding of this system. Multi-component S-layers have been described for bacteria and archaea, in which the lattice is constituted by more than one structural and functional component (Mayr et al., 1996; Veith et al., 2009; Bradshaw et al., 2017; Gambelli et al., 2019). Food for thought is how the rigid S-layer sheets are assembled on the outside of the *M. lanthanidiphila* cell. Assembly could be comparable to other S-layers that entirely adhere to the cell body. In the case of *C. crescentus*, S-layer proteins are secreted and diffuse on the lipopolysaccharide until they are incorporated at the edges of growing 2D crystals, preferentially at the cell poles and at the division site (Comerci et al., 2019). *C. difficile* and *H. volcanii* also assemble new S-layer proteins at the mid-cell during cell growth (Abdul-Halim et al., 2020; Oatley et al., 2020). Given that the *M. lanthanidiphila* S-layer sheets partially overlap each other, they could be assembled continuously, leading to individual sheets being pushed further out – perhaps until they snap off. Performing time-lapse microscopy of a growing and dividing *M. lanthanidiphila* cell might answer this question but it is currently technically impossible considering that these bacteria grow as aggregates in an enrichment culture, are anaerobic, have a slow generation time and are not genetically tractable.

Another open question regards the evolutionary advantage of the polygonal shape of *M. lanthanidiphila* (and the *Methylomirabilis* genus in general). We calculated that a *Methylomirabilis* cell with a polygonal shape has a SA:V ratio 0.2 μm-1 higher than a common rod of the same size (Supplementary Table 2). Therefore, the selective advantage of this shape for nutrient access seems limited. In laboratory enrichment cultures *Methylomirabilis* bacteria prefer to grow in aggregates with other microorganisms rather than planktonically (Gambelli et al., 2018). S-layers have been implicated to promote formation and maintenance of the aggregates (Zu et al., 2020), the flat surfaces of the cell walls of *Methylomirabilis* bacteria might aid in the attachment of one cell to the other. Moreover, flat surfaces and aggregates could help against shearing forces that these microorganisms might be exposed to in their natural habitats (Mader et al., 1999). Alternatively, predation may also be a selective factor (Beveridge et al., 1997). Indeed, the cell wall is the first element with which predators come into contact. A rigid and resilient cell wall might be harder to digest for predators, making *Methylomirabilis* bacteria a less desirable target. Finally, the possibility that the polygonal shape is not a selective trait that directly confers a survival advantage, but rather a secondary or superfluous trait, as a by-product of other selective features, cannot be excluded. Studies with knock-out mutants could unravel the molecular mechanisms and function of the polygonal cell shape. However, these techniques are currently unfeasible in the case of *Methylomirabilis* bacteria because no pure culture and genetic system are available to date. These characteristics make *Methylomirabilis* bacteria hard to manipulate and a challenging case study for cell shape investigation. Without a genetic system, a possible alternative could be to heterologously express the S-layer protein and observe if the host cell would acquire a polygonal shape.

S-layers have been a subject of investigation through electron microscopy for nearly seven decades (see Sleytr et al., 2014 for an overview). However, the recent developments in cryo-EM have sparked a renewed interest in these fascinating structures due to advancements in cryo-EM allowing us to reach (near) atomic resolution (Bharat et al., 2017; Fioravanti et al., 2019; Gambelli et al., 2019; Herrmann et al., 2020; von Kügelgen et al., 2020). Therefore, we expect additional studies that will broaden our understanding of the role of S-layers in the microbial lifestyle, and how we can exploit them as nanotechnologies.

## Data Availability Statement

The datasets presented in this study can be found in online repositories. The names of the repository/repositories and accession number(s) can be found below: ProteomeXchange, PXD029319; EMDB, EMD-13672 and EMD-13670; and EMPIAR, EMPIAR-10822 and EMPIAR-10829.

## Author Contributions

LN and MJ designed the project. LG, RM, WV, CD, AE, WE, BD, and LN designed the experiments. WV maintained the enrichment culture. WV, LG, and RM performed the enrichment of the S-layer patches. LG performed the SDS-PAGE and immunoblotting. LG and RM performed the immunogold localization. WE and CD collected the cryo-tomography data. LG, CD, and RM analyzed the cryo-tomography data with input from BD and AE. MP performed the glycosylation analysis. LN and LG wrote the manuscript with input from all authors.

## Conflict of Interest

The authors declare that the research was conducted in the absence of any commercial or financial relationships that could be construed as a potential conflict of interest.

## Publisher’s Note

All claims expressed in this article are solely those of the authors and do not necessarily represent those of their affiliated organizations, or those of the publisher, the editors and the reviewers. Any product that may be evaluated in this article, or claim that may be made by its manufacturer, is not guaranteed or endorsed by the publisher.

## Footnotes

1
https://www.thegpm.org/crap/

2
https://www.ibi.vu.nl/programs/pralinewww/

3
http://crdd.osdd.net/raghava/glycopp/

4
http://www.proteomexchange.org/
