# Supplementary material for: The Polygonal Cell Shape and Surface Protein Layer of Anaerobic Methane-Oxidizing Methylomirabilis lanthanidiphila Bacteria
Source: Front Microbiol. 2021 Dec 1;12:766527. doi: 10.3389/fmicb.2021.766527 (PMC8671808; doi:10.3389/fmicb.2021.766527)
Supplement: Supplementary file 1 [file Data_Sheet_1.PDF]

Supplementary Material

mela_00855 gene

**Gene length**	950 bp
**RPKM value**	1539.33
**Gene sequence**	ATGATTTCTGGAGCAAGGGTTCTGCGAAGGAAGAGAGTTCTGCTCTCCCTCTCAGTCTCT ATCTCGCTTGTAAGCTGGGTGCTCGTCCCAATAACCGAGGCGGGGTTCCAGTCGAACTT CTTCATGTCGAGTAACGGGACTGGCTATTTTGCTTTCAGGGATGATGGAGCAACCGGTG ATCCGGTCCAAGTCACCGGCCTCGCCCTCGGCGCCAGAAGGTCTGCTGATGGCGGCGTC AGCGCTACAACCACAGCAAACCCTGGGGACCCCCTGCAGGCTAATGTCGCAATCGACTC ACTATTCCCTGCAGCCAGTGTCCGGCACACAGGCCTTCTGAGCGGATGTACTCTCCCGG GAGATTTCAACGCCAATGCCAATGGTGGCTTTGGTAGCTGGGATGTGGGAGATGATGT GGGAGGCGTAATCTCTCTCTCAGGTAATACCCGCCTTCCAAATACCCTGAGCGGTGGAG GCGGCAACGTCCCTGCCGCGGACAACACGCTTGGGACTAAATATCGATTCGGAGGAGT CGCCCCAACAACCAGCGTCGCCTTGGGCACTGCGCTCCCTACAGACGGTATTGGGACGG CACCGGGTCTCGTGGTAATTGGCAAGTGCGTGGTGTTCGCCCTGAATAGCTCGTCGTTG CTGACCCCGGGATCGACACTTGAGGGTGCCGGCGCGGTGTCAGGGTTCCTTGCCAAAA TGGCTCCTGGTGACTCGCAGTGCGCGACTGGCACAGCATGTGCTTCCGCCGCGAAGCA GGATAATACTTCAGTATTCCTGACAACCACGACAACCACGACAACCACGACCACGACCA CGACGAGCACGACGAGCACGACAACCGTGATTCCGACGGTCGGTCCGTGGGGTATGGC GATGTTGGGTGTGGCGTTCCTGGGCGCGATGGCCTGGATGCTCAAAGCCCGCAGAGCT CACATCTCATAG

mela_00855 protein

**Protein length**	316 aa
**Domains**	1-34 aa, predicted signal peptide; 35-284 aa, non cytoplasmic domain; 285-312 aa, IPTL-CTERM protein sorting domain
**Predicted TMH**	2
**Molecular weight**	31,6 kDa
**pI**	8.42
**Automatic annotation**	hypothetical protein
**Putative *N*-linked glycosylated sites**	4
**Putative *O*-linked glycosylated sites**	46
**Protein sequence**	MISGARVLRRKRVLLSLSVSISLVSWVLVPITEAGFQSNFFMSSNGTGYFAFRDDGATGDPV QVTGLALGARRSADGGVSATTTANPGDPLQANVAIDSLFPAASVRHTGLLSGCTLPGDFNA NANGGFGSWDVGDDVGGVISLSGNTRLPNTLSGGGGNVPAADNTLGTKYRFGGVAPTTS VALGTALPTDGIGTAPGLVVIGKCVVFALNSSSLLTPGSTLEGAGAVSGFLAKMAPGDSQCA TGTACASAAKQDNTSVFLTTTTTTTTTTTTTTSTTSTTTVIPTVGPWGMAMLGVAFLGAMA WMLKARRAHIS

Supplementary Table 1. Gene and protein characteristics of the *M. lanthanidiphila* S-layer protein mela_00855. Putative N- and O-glycosylation sites are colored in purple and green respectively.

Cell				
height	1.158	um		
diameter	0.259	um		
radius	0.1295	um		
	Area	Volume	SA/V	
	2*pi*r*h	pi*r^2*h		
Cylinder	0.942232752	0.061009571	15.44402	um-1
	4*SQRT(3)*r	2*SQRT(3)*r^2*h		
Hexagon	1.038960285	0.067272678	15.44402	um-1
	4*pi*r^2	4/3*pi*r^3		
Sphere	0.210741177	0.009096994	23.16602	um-1
Hexagonal pyramid	2*SQRT(6)*r^2	2*r^3/SQRT(3)		
	0.082157111	0.002507718	32.7617	um-1
Cylinder+Sphere	1.152973929	0.070106565	16.44602	um-1
Hexagon+2*hexagonal pyramid	1.203274506	0.072288114	16.64554	um-1

Supplementary Table 2. Calculation of SA:V ratio of a hexagonal prism-shaped cell and a rod-shaped cell of the same size.

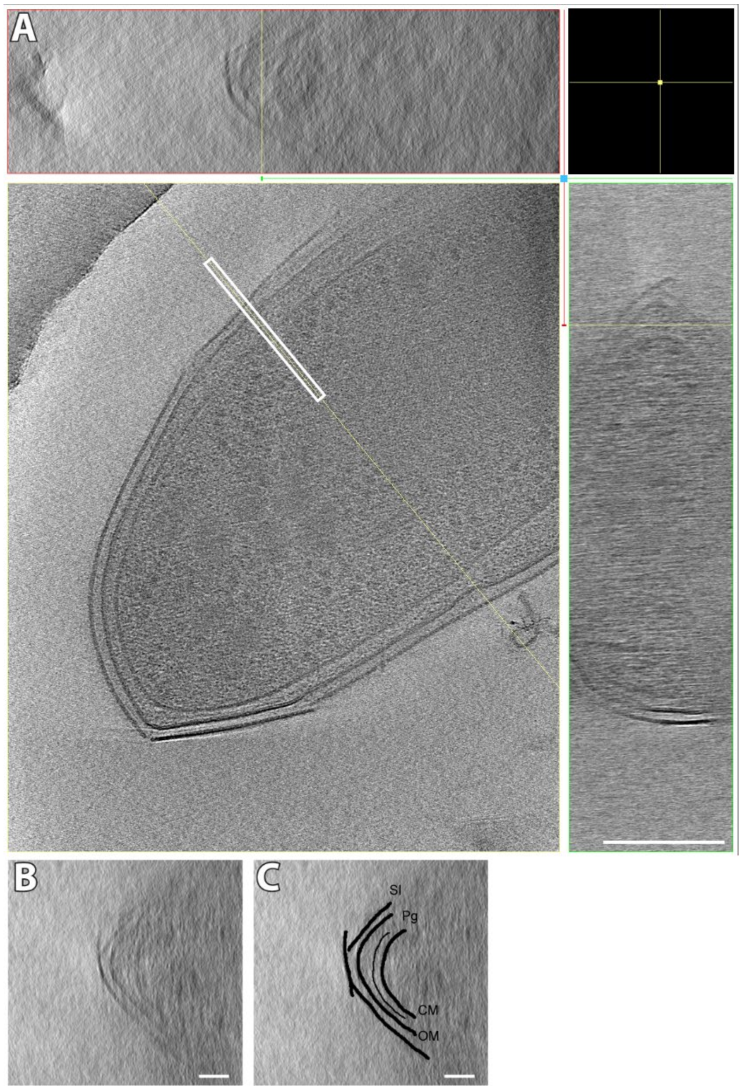

Supplementary Figure 1. Reconstructed cryo-tomogram of *M. lanthanidiphila*. The XYZ view (**A**) shows the central slice in the XY plane with the corresponding XZ (top) and XY (right) cross-sections of the reconstructed volume. In a cross-section of the reconstructed volume (**B**, segmentation in panel **C**), taken at the white rectangle in panel A, the S-layer (Sl) sheets, outer membrane (OM), peptidoglycan (Pg) and cytoplasmic membrane (CM) can be clearly observed. Note that the OM follows the sharper edges of the Sl, while the Pg follows a smoother curve. Scalebar 200 nm in panel A, 50 nm for panels B and C. XY, XZ and cross section planes are the summation of 20 consecutive virtual slices.

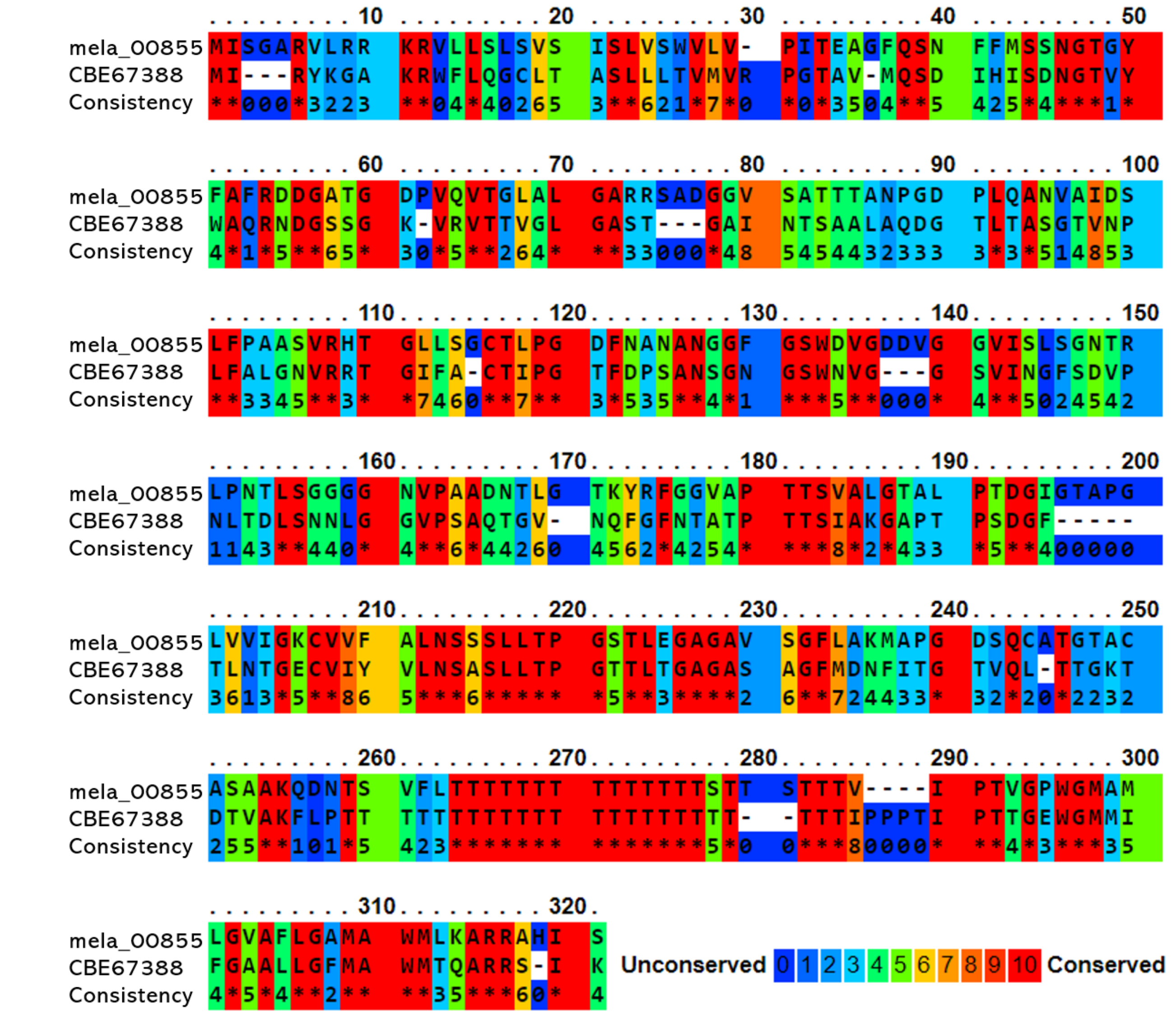

Supplementary Figure 2. Sequence alignment comparing the S-layer protein of *M. lanthanidiphila*, mela_00855, and the putative S-layer protein of *M. oxyfera*, NCBI ID: CBE67388. The sequence identity is 40.88% and sequence coverage is 97%. Sequence alignment performed with PRALINE.

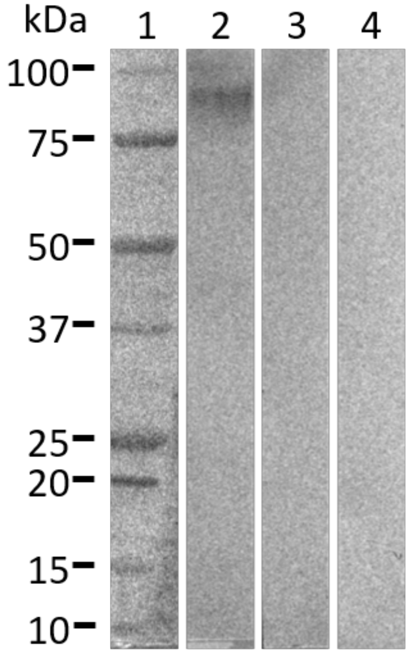

Supplementary Figure 3. Immunoblot analysis confirmed the affinity and specificity of the mela_00855 antiserum using *M. lanthanidiphila* crude cell extract. The immunoblot was performed on the crude cell extract of *M. lanthanidiphila* using crude antiserum targeting the mela_00855 (S-layer) protein. The 4-15% SDS-PAGE gel was loaded with 20 µg protein/lane (*M. lanthanidiphila* crude cell extract) and blotted onto a nitrocellulose membrane. Lane 1: marker; lane 2: incubation with crude antiserum diluted 1000-fold; lane 3: incubation with pre-immune serum diluted 125-fold; lane 4: incubation with secondary antibody only. Lanes 3 and 4 are negative controls. The empty upper part of the blot was cropped in the final image. The antiserum was tested at different dilutions (125-, 500-, and 1000-fold) until a single, dominant band was present on the immunoblot. A dilution of 1000-fold was determined as high enough to prevent a-specific binding while still enabling specific binding to the antigen. The immunoblot (2: 1000-fold diluted antiserum) showed a band around 90 kDa. No bands were visible in the two negative controls. Because the monomeric size of the S-layer protein mela_00855 is 31.6 kDa, the bands at ~90 kDa may represent a multimeric form of the S-layer protein (with potentially, additional modifications).

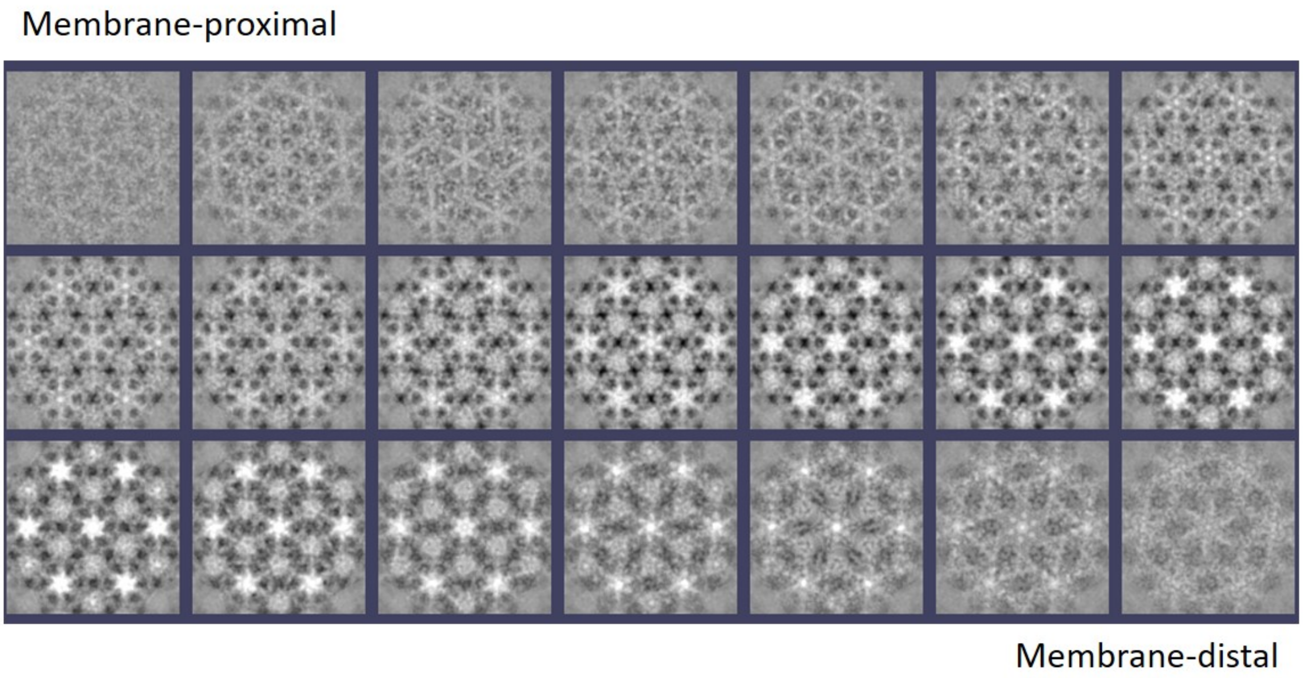

Supplementary Figure 4. Consecutive slices of the sub-tomogram averaging map of the *M. lanthanidiphila* S-layer shown in fig 4. Z step = 2.

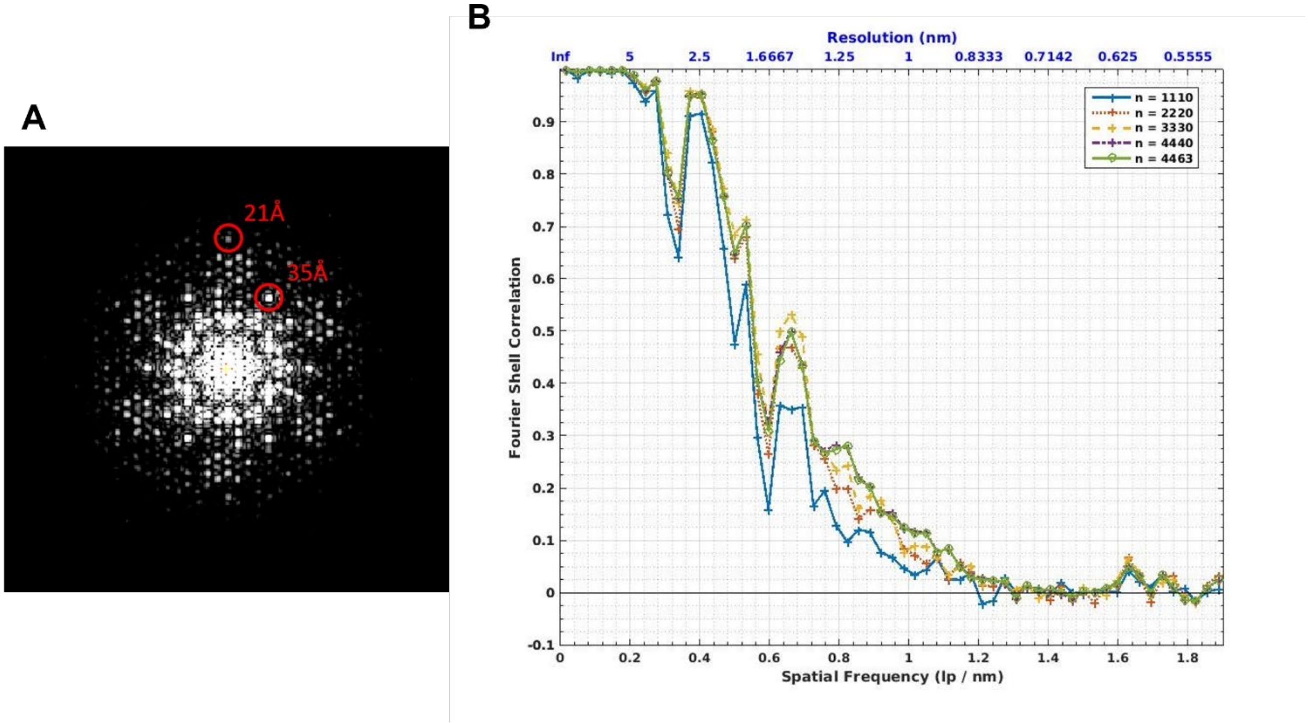

Supplementary Figure 5. (**A**) Resolution of the *M. lanthanidiphila* S-layer sub-tomogram average determined by FFT, and (**B**) FSC as obtained from Peet.

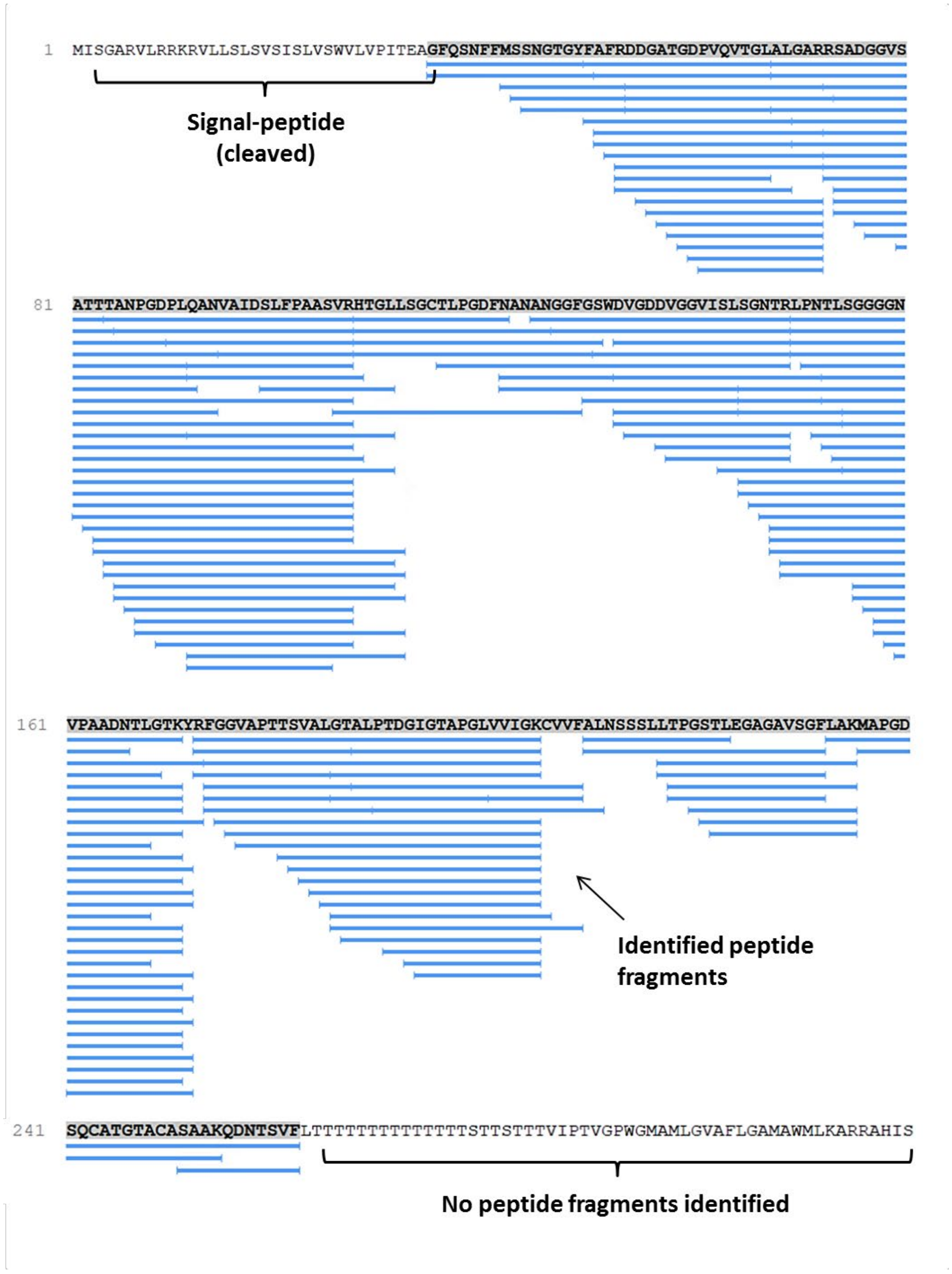

Supplementary Figure 6. The above image outlines the obtained amino acid sequence coverage for the enriched S-layer protein mela_0855, as obtained after proteolytic digestion (Trypsin, or Chymotrypsin), and shotgun proteomic analysis. The image shows the combined coverage obtained from both enzymes. The N-terminal signal peptide is cleaved and therefore not detected. A large number of (unmodified) peptide fragments were detected across the complete S-layer protein, except for the C-terminal tail, which could not be detected in this analysis.

Supplementary movie 1 and 2. Reconstructed tomograms of the two *M. lanthanidiphila* cells in fig. 1A and B respectively.

Supplementary movie 3. Sub-tomogram averaging of isolated *M. lanthanidiphila* S-layer.
